# Supplementary material for: Preclinical validation of an Escherichia coli O-antigen glycoconjugate for the prevention of serotype O1 invasive disease
Source: Microbiol Spectr. 2024 May 3;12(6):e04213-23. doi: 10.1128/spectrum.04213-23 (PMC11237799; doi:10.1128/spectrum.04213-23)
Supplement: Supplemental material — Tables S1 and S2; Fig. S1-S7. [file spectrum.04213-23-s0001.pdf]

Supplemental Table S1:

Isolate			Genotype				Phenotype	
ID	Date, State	Patient Age	LPS Core	K-antigen	MLST CC	Plasmid	anti-O1a WB	Resistance
PFEEC0074	2013, NE	-1	R4	NT	38	pS88	OAg+	
PFEEC0418	2015, CO	30	NT	NT	38		Negative	FQ, ESBL, Tc, Gm
PFEEC0134	2014, CA	73	R1	K1	59		OAg+	
PFEEC0174	2014, OH	0	R1	K1	59		OAg+	
PFEEC0169	2014, NY	71	R4	NT	648		OAg+	
PFEEC0195	2014, TX	68	R4	K20	648		OAg+	FQ, ESBL
PFEEC0475	2016, CO	67	R4	NT	648		OAg+	ESBL
PFEEC0308	2015, WI	53	R4	K20	648		OAg+	FQ, TC
PFEEC0120	2014, CO	55	R1	K1*	95	pS88	OAg+	
PFEEC0121	2014, CO	28	R1	K1*	95	pS88	OAg+	
PFEEC0122	2014, CO	43	R1	K1	95		OAg+	
PFEEC0127	2014, CO	61	R1	K1	95		OAg+	
PFEEC0148	2014, CA	64	R1	K1	95		Negative	
PFEEC0171	2014, OH	0	R1	K1*	95	pS88	OAg+	
PFEEC0175	2014, OH	0	R1	K1	95		Negative	
PFEEC0182	2014, MI	86	R1	K1	95		OAg+	
PFEEC0186	2014, NY	54	R1	K1	95		OAg+	
PFEEC0189	2014, KS	59	R1	K1	95		OAg+	
PFEEC0203	2014, TX	49	R1	K1	95		OAg+	
PFEEC0223	2014, KS	-1	R1	K1	95		OAg+	
PFEEC0225	2014, KS	-1	R1	K1	95		OAg+	
PFEEC0245	2014, WA	48	R1	K1*	95		OAg+	TIM
PFEEC0311	2015, WI	56	R1	K1	95		Negative	
PFEEC0070	2013, ND	85	R1	K1	95		Negative	
PFEEC0085	2013, NE	-1	R1	K1*	95	pS88*	OAg+	
PFEEC0089	2013, NE	-1	R1	K1*	95	pS88*	OAg+	
PFEEC0264	2014, CO	63	R1	K1	95		OAg+	
PFEEC0280	2014, MA	0	R1	K1	95		OAg+	
PFEEC0283	2014, MA	0	R1	K1	95		OAg+	
PFEEC0410	2015, CO	66	R1	K1	95		Negative	Gm
PFEEC0435	2015, NY	71	R1	K1*	95		OAg+	
PFEEC0303	2014, IL	64	R1	K1	95		OAg+	
PFEEC0346	2015, WI	30	R1	K1	95		OAg+	
PFEEC0460	2016, OH	0	R1	K1	95		OAg+	
PFEEC0480	2016, CO	52	R1	K1	95		OAg+	FQ, ESBL
PFEEC0517	2015, FL	4	R1	K1*	95	pS88	OAg+	
PFEEC0440	2015, NY	56	R1	K1	95		OAg+	
PFEEC0387	2015, OH	77	R1	K1	95		OAg+	
PFEEC0394	2015, ND	79	R1	K1	95		OAg+	
PFEEC0399	2015, NY	67	R1	K1*	95		OAg+	TIM
PFEEC0497	2016, IN	85	R1	K1*	95	pS88	OAg+	

Table S1. U.S. *E. coli* Serotype O1 blood isolates
Abbreviations: NT, non-typeable; MLST CC, Multi-Locus Sequence Type Clonal Complex; WB, Western immunoblot Blot of phenol extracted LPS (run on SDS-PAGE gels) probed with rabbit anti-serotype O1a antisera (supplemental Fig. S6); FQ, fluoroquinolone; ESBL, extended-spectrum β-lactamase (cephalosporin); Tc, tetracycline; Gm, gentamycin; TIM, Ticarcillin Clavulanic Acid; pS88*, sequences of recovered pS88 plasmids, available as Genbank IDs OP331339 and OP331340; K1*, capsule genotype confirmed by flow cytometry with mAb 13D9-151.

Supplemental Figure S1:

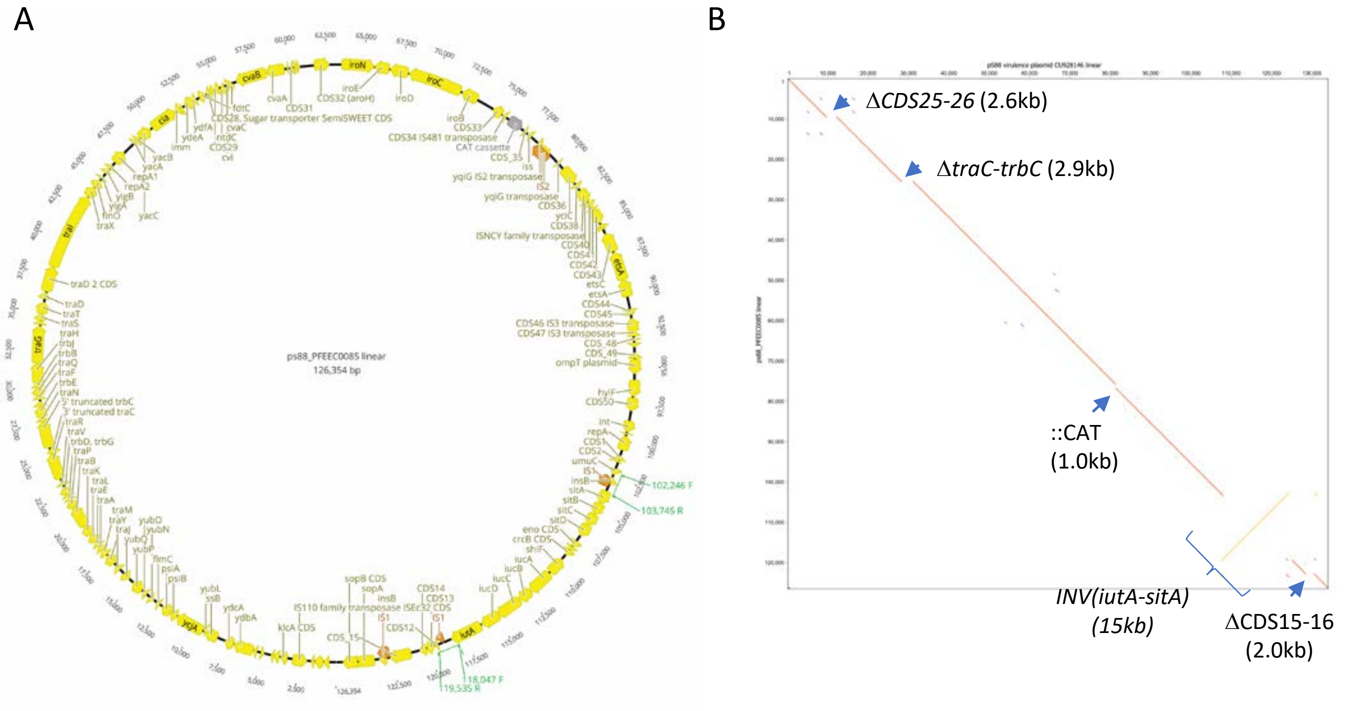

Fig. S1. Structure of the pS88-like plasmid in isolate PFEEC0085. (A) annotated map of the nucleotide sequence available as GenBank accession OP331340.1. (B) Identity dot matrix comparison of the 126kb sequence with CAT insertion (OP331339) compared with the 133kb pS88 reference (CU928416). Discontinuous breaks representing deletions, a targeted CAT insertion and an inversion are highlighted. Images were generated with Geneious Prime ® 2020.1.2 (created by Biomatters). The panel B dot matrix used default settings (global alignment with free end gaps and cost matrix of 65% similarity). Three plasmid deletions relative to this reference were identified: a 2.6kb deletion in CDS25-CDS6 (IS21 transposase orfs 1 and 2), a 2.9 kb deletion spanning *traC-trbC* and a 2.0 kb deletion of CDS15-16 (unknown function). Finally, a 15.5 kb region between two IS1 elements containing *sitA-D* and *iuCA-iutA* virulence gene clusters was found to be inverted. These same sequence differences were confirmed to also be present in total genome sequence data from parental isolate PFEEC0085, indicating that these sequence differences were not a consequence of plasmid manipulation. The presence of the gene deletion in transfer (*tra*) genes, as well as a stop codon in codon 133 of *traD* in the plasmid from this strain explained our inability to mobilize it by conjugal transfer into a streptomycin resistant ST95 O1:K1:H7 recipient strain PFEEC0435, which lacks the megaplasmid.

Supplemental Figure S2:

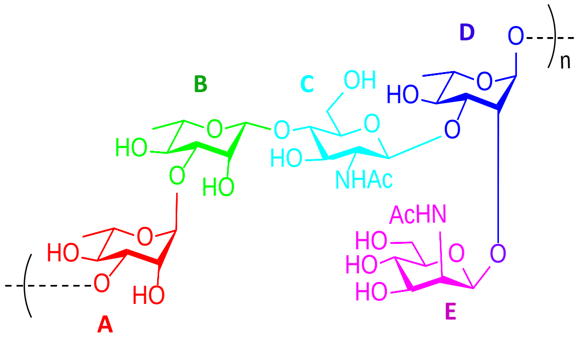

-3)-α-L-Rha*p*-(1→3)-β-L-Rha*p*-(1→4)-β-D-Glc*p*NAc-(1→3)-α-L-Rha*p*-(1-
2
|
β-D-Man*p*NAc-(1-

		PFEEC0007		Literature Assignment	
		1H (ppm)	13C (ppm)	1H (ppm)	13C (ppm)
α-L-Rha*p* (A)	A1	5.02	102.4	4.96	103.2
	A2	4.11	70.3	4.07	71.1
	A3	3.89	78.6	3.85	79.4
	A4	3.55	71.8	3.49	72.6
	A5	3.88	69.6	3.86	70.4
	A6	1.29	17.0	1.28	17.9
β-L-Rha*p* (B)	B1	4.89	100.8	4.85	101.6
	B2	4.15	70.9	4.10	71.7
	B3	3.63	80.7	3.58	81.5
	B4	3.48	71.6	3.43	72.3
	B5	3.42	72.5	3.73	73.3
	B6	1.33	17.0	1.20	17.9
β-L-Glc*p* NAc (C)	C1	4.79	102.3	4.73	103.2
	C2	3.74	56.6		57.3
	C3	3.74	74.0		74.8
	C4	3.69	77.5		78.2
	C5	3.54	75.0		75.8
	C6-1	3.96	61.5		61.7
	C6-2	3.85	61.5		61.7
	CH_3	2.05	22.7		23.4
	CO		175.0		175.8
α-L-Rha*p* (D)	D1	5.17	101.6	5.12	102.5
	D2	4.35	77.4	4.30	78.1
	D3	3.97	79.5	3.92	80.3
	D4	3.47	71.6	3.41	72.5
	D5	3.80	69.7	3.81	70.5
	D6	1.25	17.1	1.24	17.8
β-D-Man*p* NAc (E)	E1	5.04	100.2	5.00	101.1
	E2	4.55	53.5	4.50	54.3
	E3	3.80	72.5	3.76	73.3
	E4	3.52	67.3	3.46	69.3
	E5	3.36	76.6	3.32	77.3
	E6-1	3.86	61.0		62.2
	E6-2	3.81	61.0		62.2
	CH_3	2.07	22.5		23.2
	CO		175.6		176.4

Fig. S2. ^{1}H and ^{13}C NMR signal assignment of O1a long-chain O-antigen from PFEEC0007 (ST59) and comparison of chemical shifts reported in literature (Jann et al 1992. Carbohydrate polymers 18:51-57). There are some missing proton assignments in this publication.

Supplemental Figure S3:

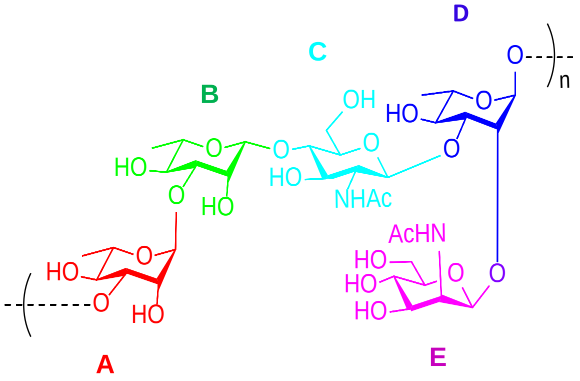

E. coli O1a PFEC0007 vs E. coli O1a PFEC0497

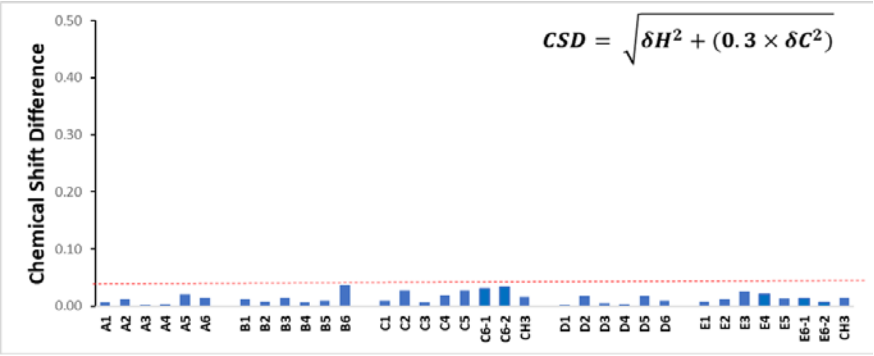

E. coli O1a PFEC0007 vs E. coli O1a PFEC0074

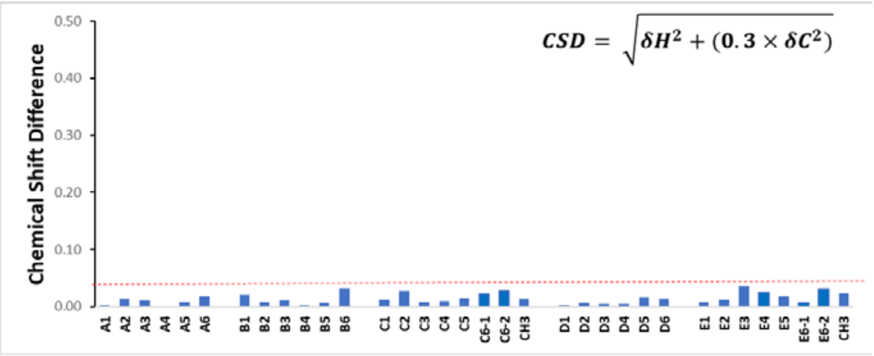

Fig. S3. Comparison of NMR chemical shifts between isolate PFEEC0074, *PFEEC0497* and PFEEC0007. Differences are within the tolerance limit, which indicates that the native *E. coli* O1 PFEEC0074 and PFEEC0497 structure matches well the *E. coli* O1 long chain PFEEC0007.

Supplemental Figure S4:

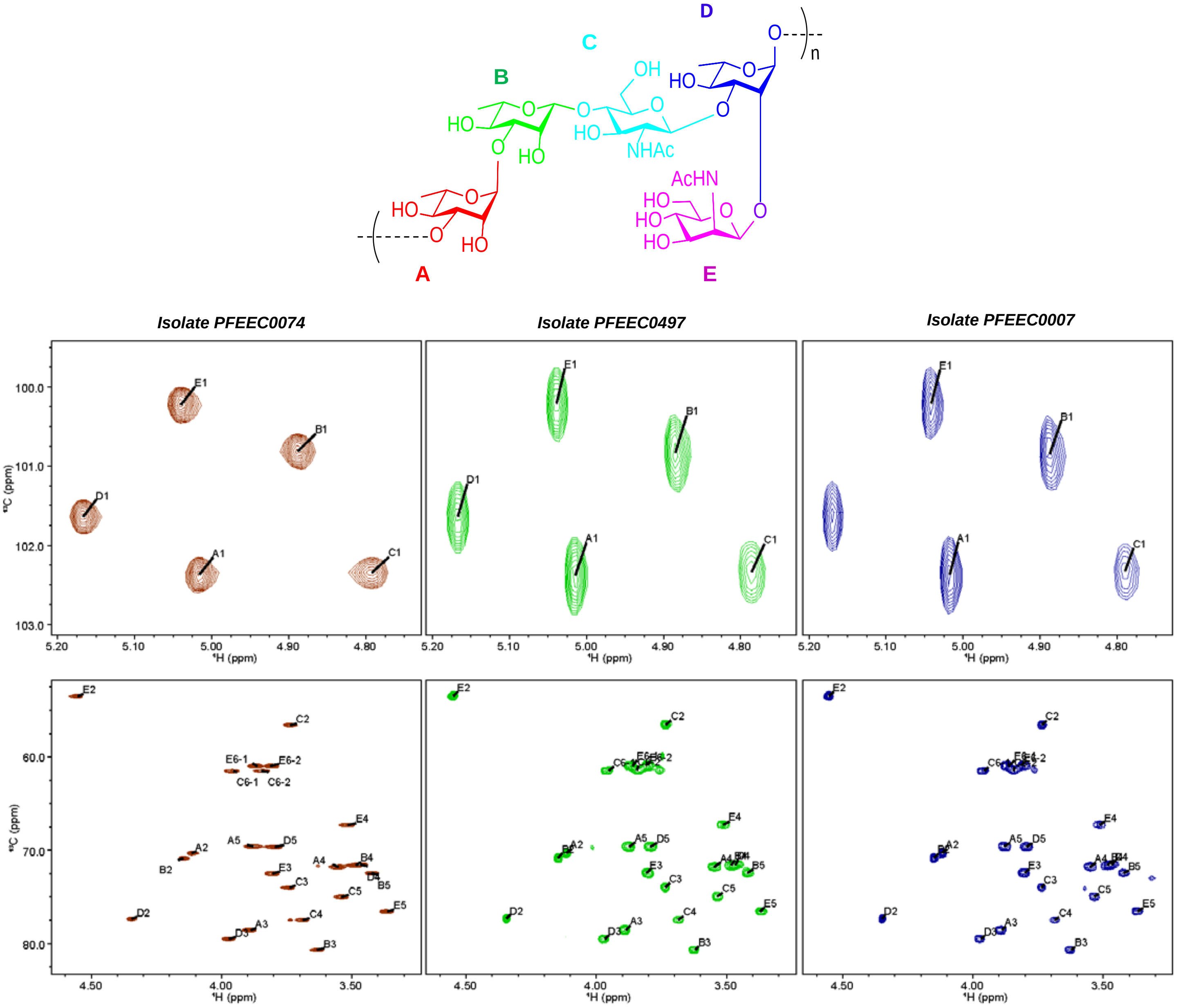

Fig. S4. Expanded ^{1}H-^{13}C HSQC spectra showing the anomeric and ring resonances of *E. coli* O1a polysaccharide produced from PFEEC0074 (ST38) (brown), PFEEC0497 (ST95) (green) and PFEEC0007 (ST59) *wzzB/fepE* (blue). All peaks are annotated. There are no structural difference seen between these polysaccharides.

Supplemental Figure S5:

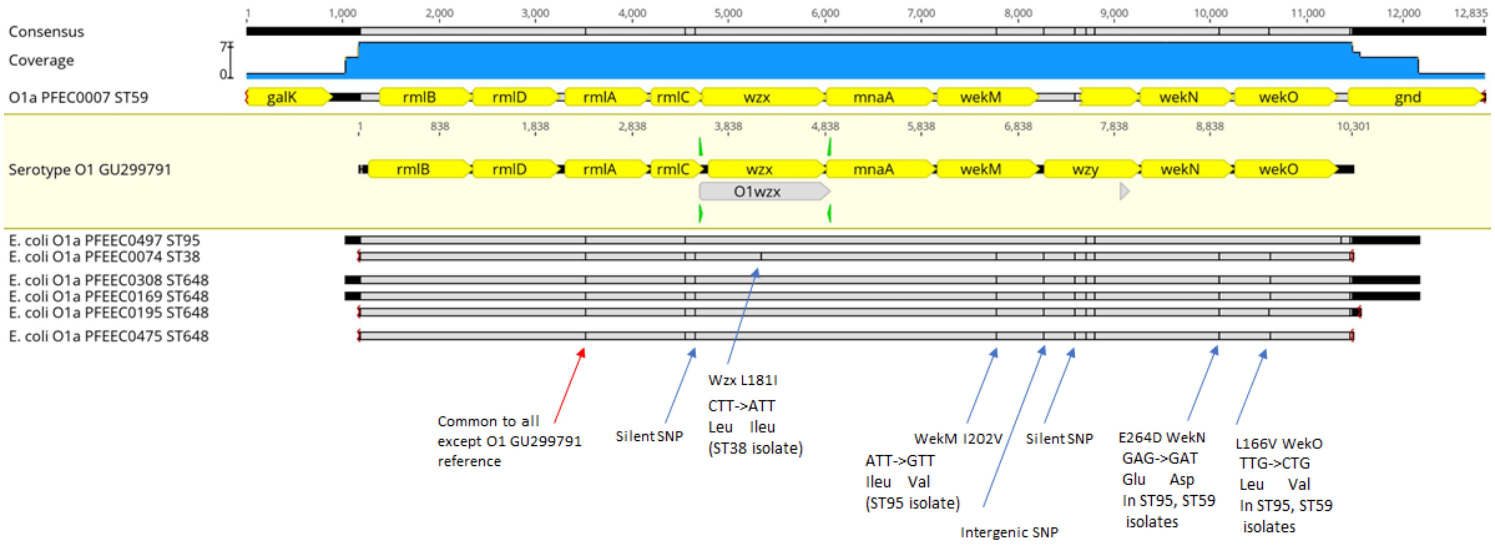

Fig. S5 *E. coli* O1a O-antigen operon single nucleotide polymorphisms (SNPs). Gene clusters were extracted from assembled WGS contigs and aligned with reference operon Genbank GU299791 using Geneious Prime® Software v202.1.2. The O1a gene cluster spans 10.05 kb and ten genes located between *galK* and *gnd* anchor genes. Tick marks and arrows identify SNP differences between ATLAS strain sequences and the reference. Gene clusters from the four ST648 O1 isolates share 100% nucleotide identity. Conservative substitutions in *wzx*, *wek*M, *wek*N and *wek*O genes are highlighted relative to ST59, ST95 and ST38 isolates for which O-antigen repeat structures were confirmed by NMR.

Supplemental Figure S6:

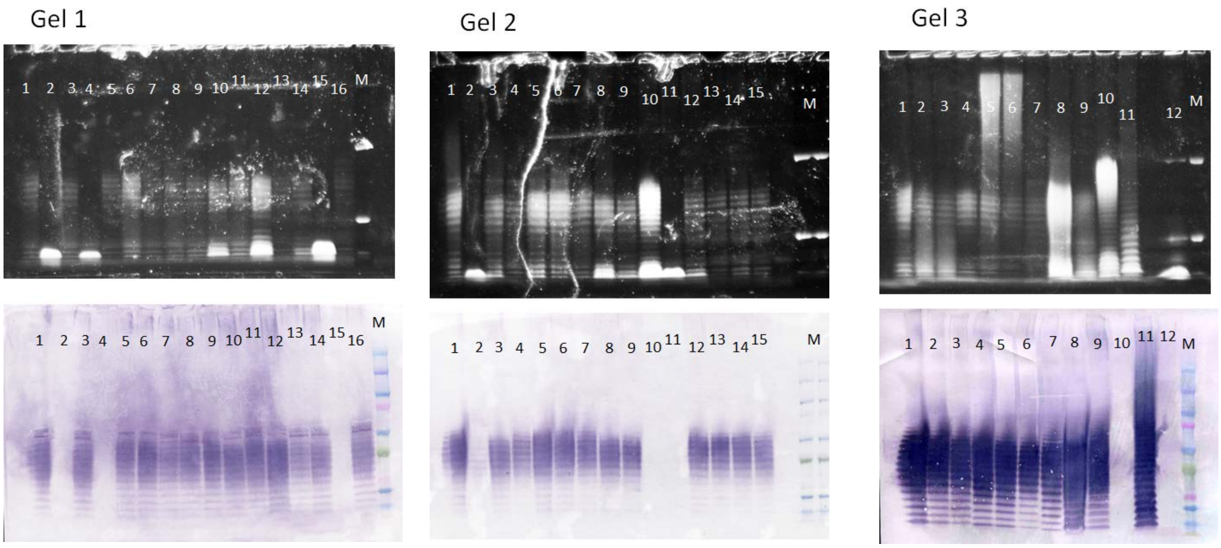

1. PFEEC0435
2. PFEEC0435 *waaL* negative control
3. PFEEC0225
4. PFEEC0148 ΔO1a LPS
5. PFEEC0394
6. PFEEC0346
7. PFEEC0127
8. PFEEC0189
9. PFEEC0186
10. PFEEC0223
11. PFEEC0122
12. PFEEC0480
13. PFEEC0121
14. PFEEC0085
15. PFEEC0085 *waaL* negative control
16. PFEEC0399

M. Kaleidoscope™ marker (Biorad)

1. PFEEC0460
2. PFEEC0175 ΔO1a LPS
3. PFEEC0283
4. PFEEC0280
5. PFEEC0303
6. PFEEC0387
7. PFEEC0440
8. PFEEC0182
9. PFEEC0264
10. PFEEC0410 unrelated O-antigen
11. PFEEC0070 ΔO1a LPS
12. PFEEC0203
13. PFEEC0120
14. PFEEC0517
15. PFEEC0245

M. Kaleidoscope™ marker (Biorad)

1. PFEEC0497
2. PFEEC0171
3. PFEEC0089
4. PFEEC0475
5. PFEEC0308
6. PFEEC0195
7. PFEEC0169
8. PFEEC0174
9. PFEEC0134
10. PFEEC0418 unrelated O-antigen
11. PFEEC0074
12. PFEEC0311 ΔO1a LPS

M. Kaleidoscope™ marker (Biorad)

Fig. S6 SDS-PAGE profiles of LPS extracted from ATLAS BSI serotype O1 strains. LPS was purified from 2 mL overnight LB cultures using a phenol extraction kit (Bulldog Bio). Sample loadings spanned three 4-12% acrylamide SDS-PAGE gels (Biorad Criterion) run in duplicate in MOPS buffer. One gel was stained with a Pro-Q Emerald Green-3000 kit (ThermoFisher), the other was blotted via electrophoretic transfer to PVDF membrane and probed for 1h at RT with a 1:2,000 dilution of rabbit O1a glycoconjugate immune sera after blocking with 5% milk in TBS 0.1% tween®20 detergent. After washing in TBS tween buffer, membrane were incubated with a 1:5,000 dilution of secondary Goat anti-rabbit HRP antibody (Southern Biotech) for 1h RT followed by colorimetric detection (HRP Kit, ThermoFisher). LPS from 85% of strains cross-reacted with the O1a antisera. Four strains have truncated LPS that migrate with LPS from control O-antigen minus *ΔwaaL* mutants. Two strains express O-antigens that fail to cross react with the immune sera.

Supplemental Figure S7

A.

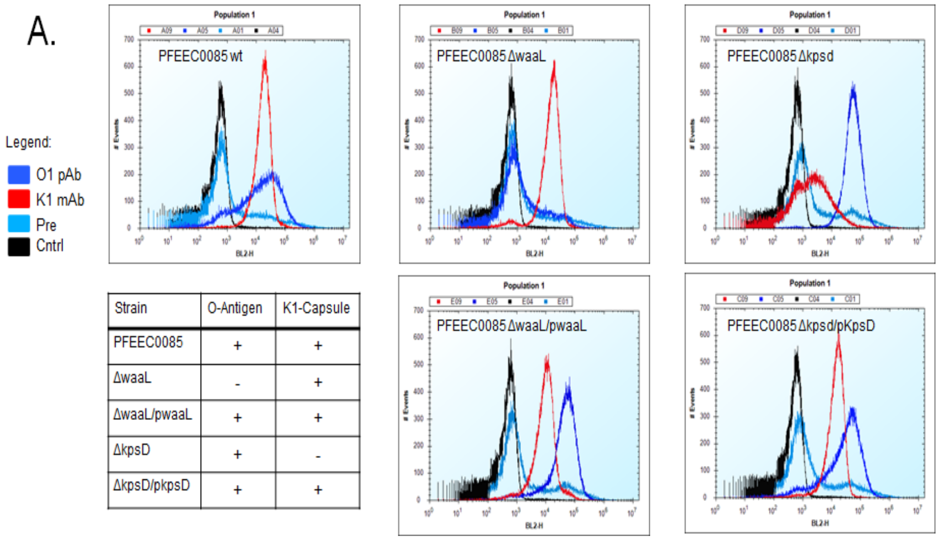

Strain	O-Antigen	K1-Capsule
PFEEC0085	+	+
ΔwaaL	-	+
ΔwaaL/pwaaL	+	+
ΔkpsD	+	-
ΔkpsD/pkpsD	+	+

B.

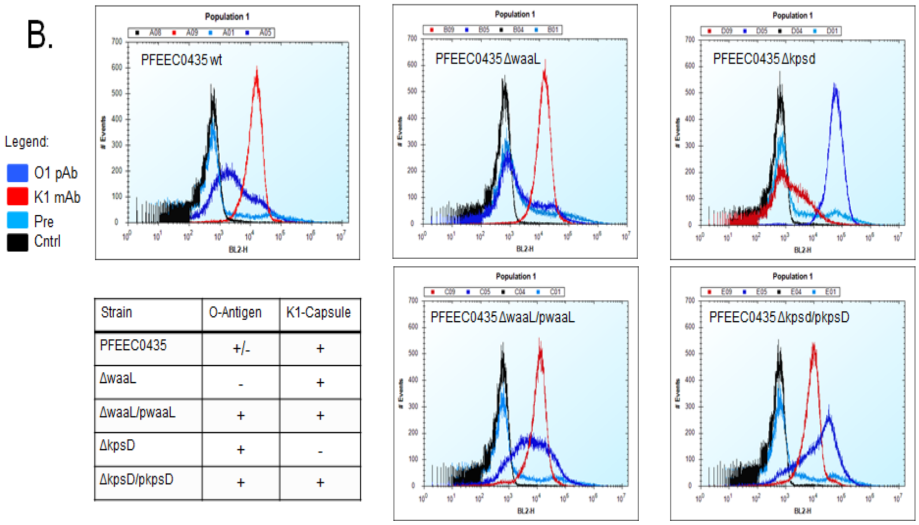

Strain	O-Antigen	K1-Capsule
PFEEC0435	+/-	+
ΔwaaL	-	+
ΔwaaL/pwaaL	+	+
ΔkpsD	+	-
ΔkpsD/pkpsD	+	+

Fig. S7. Surface expression of the serotype O1 O-antigen and K-capsule in isogenic O1:K1:H7 isolates grown in LB media to stationary phase (OD_{600} of 2.0). These conditions favor high-level K-capsular antigen expression that partially masks underlying O-antigen. Flow cytometry profiles of PFEEC0085 (A) and PFEEC0435 (B) and their derived mutants. Introduction of *waaL* and *kpsD* complementing plasmids into respective O-antigen and K-capsular polysaccharide null mutants was sufficient to restore K-antigen and O-antigen surface expression. O1 pAb, 1:100 immune rabbit sera; K1 mAb, 4µg/ mL mAb 13D9-151; Pre, pre-immune rabbit sera; Cntrl, secondary antibody only.

Supplemental Table S2:

Timepoint	Antigen/ dose	IgG GMT (μg/mL) (95% CI)	OPA GMT (95% CI)	OPA responder rate
PD2	PBS	0.002 (0.002, 0.004)	50	0%
PD3	PBS	0.002 (0.002, 0.002)	50	0%
PD2	2μg free poly	0.003 (0.002, 0.006)	50[ǂ]	-
PD3	2μg free poly	0.002 (0.002, 0.003)	50[ǂ]	-
PD2	0.2μg conjugate	0.324 (0.091, 1.16)	141 (53, 377)	25%
PD3	0.2μg conjugate	3.65 (0.84, 15.9)	1,173 (341, 3,916)	70%
PD2	2μg conjugate	4.47 (1.61, 12.4)	730 (195, 2,735)	60%
PD3	2μg conjugate	17.8 (8.1, 39.1)	6,076 (2,085, 1,7707)	90%

[ǂ]OPA titers determined from serum pools

Table S2. Serotype O1a glycoconjugate immunogenicity in mice (Summary of IgG and OPA GMTs in Figure 5 B-C plots).
